# Supplementary material for: Information processing in patterned magnetic nanostructures with edge spin waves
Source: Sci Rep. 2017 Jul 17;7:5597. doi: 10.1038/s41598-017-05737-8 (PMC5514091; doi:10.1038/s41598-017-05737-8)
Supplement: Supplementary file 5 — Supplementary material [file 41598_2017_5737_MOESM5_ESM.pdf]

Information processing in patterned magnetic nanostructures with edge spin waves

Antonio Lara[1], Javier Robledo[1], Konstantin Y. Guslienko[2] and Farkhad G. Aliev[1]

[1]Dpto. Física Materia Condensada, C03, Universidad Autónoma de Madrid, 28049, Madrid, Spain
[2]Dpto. Física de Materiales, Universidad del País Vasco, UPV/EHU, 20018 San Sebastián, Spain and IKERBASQUE, the Basque Foundation for Science, 48013 Bilbao, Spain

May 10, 2017

Abstract

This part describes the Supplementary Materials.

Variation of vortex state annihilation field with dot size

When thinking about creation of real devices one could wonder, how small could triangular dots be in practice to be able to carry edge spin waves in reasonably small bias fields. Related point to consider when dealing with small dots is that the vortex state gets more difficult to annihilate, and threfore higher fields are necessary to reach saturation. Such change in vortex annihilation field is presented in the simulated hysteresis loops shown and analysed in Supplementary Figure 1.

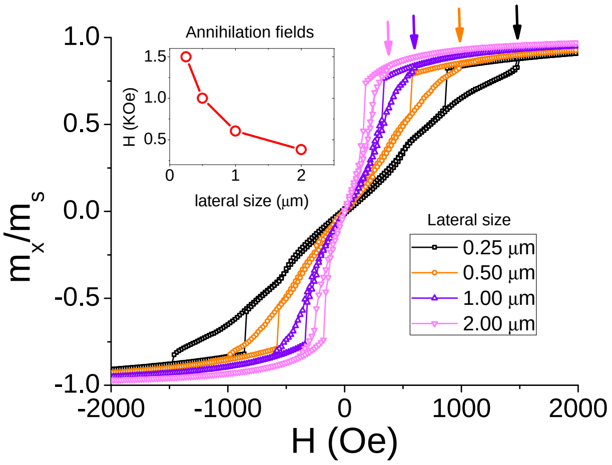

Figure 1: Simulated magnetization loops in $100nm$ thick equilateral triangular Py dots of different lateral size. Insert shows how vortex anihillation field changes with lateral size

Edge domain wall profiles in triangles and rectangles

In the main text we consider the case of patterned magnetic elements - equilateral triangular magnetic dots with sides of length 2000 nm and thickness 30 nm. In the Buckle (B) state the static bias magnetic field H_{DC} is directed along the triangle base. This geometry avoids the "butterfly" type edge domain walls (E-DWs) which appear for fields applied completely perpendicular to the base of triangule (Y state). This type of domain wall, with a magnetic discontinuity in the middle, due to geometry also appears in rectangular or square shapes when the field is parallel to two faces and, therefore, perpendicular to the other two (Figure 2 in the main text).

Previous studies of the spin waves in rectangular magnetic elements and long strips only used in-plane high frequency fields perpendicular to the applied DC field. Then, increasing the DC field perpendicular to the edge affects the E-DW related energy so that mainly SWs are excited in the center of the strip [1]. Even if there is some splitting of the main modes into two modes that propagate closer to the sides, the perpendicular alignment of DC field and high frequency field does not excite spin waves at the edges, due to the zero torque of this excitation on the spins at the edge, which lay parallel to it, in order to reduce the magnetostatic energy outside the dot. In Figure 3 in the main text we show a simulation of the corresponding effective field profiles.

Spectrum of edge spin waves under DC field at different angles

Supplementary Figure 2 shows the amplitude of the FFT spectra of a $1\mu m$ triangular dot after being excited with a short gaussian field pulse. The different spectra are simulations with the DC field applied at different angles with respect to the horizontal, 0, 4 and 8 degrees. The spectra shift in frequencies (not much, but still a noticeable change) with the angle. This angle controls the magnetic state, passing from a B state at 0° to a Y state at 90°, with intermediate states in between (left part, showing the exchange energy density for 0 and 20°). If a fine tuning of the eigenmode frequencies is needed, a field perpendicular to the main DC field, in order to produce a tilted field, can do the job.

Influence of magnetic field on formation of edge domain walls in smaller dots

The size of the triangles is important for edge spin wave transmission. In the limit of large dots, the main hurdle to

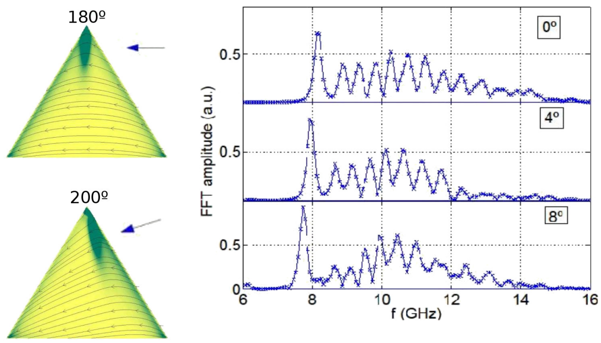

Figure 2: Left panels show the exchange energy distribution for a horizontal (top) applied field and for a 20º inclined with respect to the horizontal field (bottom). The right panel shows how eigenfrequencies are shifted even for slightly different applied field angles.

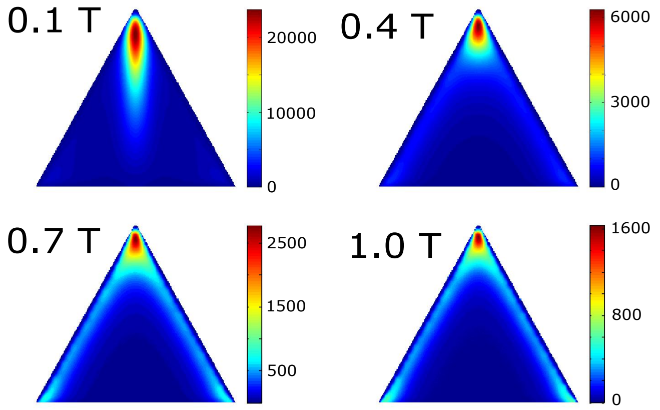

Figure 3: Exchange energy density in a 0.25 μm triangular dot for different applied fields, indicated in T next to each dot. The color scale represents the exchange energy density, in J/m^3.

overcome is wave dissipation due to damping, whereas for smaller dots it is due to the overlap that naturally occurs between the exchange channels when the size of the dot is decreased. A similar situation occurs always, for example, in the top vertex of a dot in the B state, with the field applied parallel to the base, opposite to this vertex. There, at the vertex, both exchange channels meet and become a unique magnetic charge. The only option to keep the exchange channels separate in a smaller dot is to apply a stronger magnetic field, to confine the channels closer to the edges. As this is done, the excess of exchange energy stored in the channel decreases, as shown in Supplementary Figure 3.

Triangular dots under an exchange bias field

As mentioned in the main text, it is not necessary to have an external field which saturates the sample to benefit from the properties of the B or Y states. Supplementary Figure 4 shows simulations of hysteresis loops of a 2 μm long, 30 nm thick triangular dot placed on top of a layer of fixed spins,

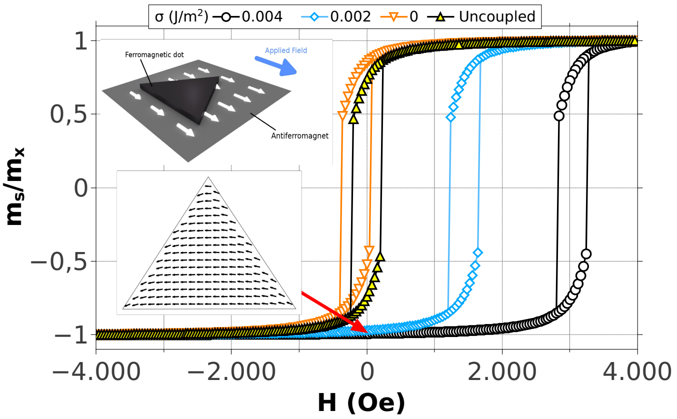

Figure 4: Simulated hysteresis loops for a triangular dot in the B state with different values of exchange bias due to the coupling to an antiferromagnet.

which represents an antiferromagnet. Different exchange couplings between the two layers shift the hysteresis cycle to higher or lower fields, so that be B or the Y state could be achieved at zero external field using this method. The saturated state, with edge domain walls ready for propagating spin waves can be achieved in the absence of an external field.

References

[1] Vladislav E. Demidov, Sergej O. Demokritov, Karsten Rott, Patryk Krzysteczko, and Guenter Reiss. Nano-optics with spin waves at microwave frequencies. *Applied Physics Letters*, 92(23), 2008.

Video 1: Excitation of edge spin waves in a 30 nm thick, 2 μm in lateral length triangular dot in the B state. The excitation source is an ac magnetic field applied to the lower left corner, directed in the horizontal direction, with a frequency f=12.383 GHz (one of the many eigenmode frequencies for a DC field of 1000 Oe). The waves propagate with the highest intensity along the edges, and once they reach the upper vertex, they are redirected to the lower right corner. The colors represent the changes in mx from the saturation magnetization.

Video 2: Excitation of both lower vertices of a 30 nm thick, 1 μm in lateral length triangular dot in the B state. Both vertices are excited in phase and the waves sum in the upper vertex. The excitation frequency of both sources is f=11.259 GHz.

Video 3: Same as video 2, but the excitations are in opposition of phase, producing a different type of interference in the upper vertex.

Video 4: Example simulation of a "splitter", composed of two 1 μm long triangular dots, linked by their bases, with a slit between the two, and the rhomboid resulting structure has attached two strips to carry spin waves in and out. The whole structure is 30 nm thick. Spin waves are excited locally at one end of the strips, split in half when they reach the first triangular shape and are able to overcome the slit, to be joined again together at the second strip, to generate spin waves through it. Long distance dipolar waves are generated as well, but the edge spin waves propagate exclusively next to the borders of the structure and don't almost reflect at the slit. The excitation frequency is f=12.73 GHz. The waves get reflected back when they reach the end of the second strip, but if it were longer, or this device was connected to another, this wouldn't happen. The field is applied horizontal, so the triangular parts are in the B state.
